# Early detection of changes in phospholipid metabolism during neoadjuvant chemotherapy in breast cancer patients using phosphorus magnetic resonance spectroscopy at 7T

**DOI:** 10.1002/nbm.4086

**Published:** 2019-03-29

**Authors:** Erwin Krikken, Wybe J.M. van der Kemp, Paul J. van Diest, Thijs van Dalen, Hanneke W.M. van Laarhoven, Peter R. Luijten, Dennis W.J. Klomp, Jannie P. Wijnen

**Affiliations:** ^1^ Department of Radiology University Medical Center Utrecht Utrecht The Netherlands; ^2^ Department of Surgery, Diakonessenhuis Utrecht The Netherlands; ^3^ Department of Medical Oncology, Academic Medical Centre Amsterdam Amsterdam The Netherlands

**Keywords:** 7 T, 31P‐MRSI, breast imaging, high field

## Abstract

The purpose of this work was to investigate whether noninvasive early detection (after the first cycle) of response to neoadjuvant chemotherapy (NAC) in breast cancer patients was possible. ^31^P‐MRSI at 7 T was used to determine different phosphor metabolites ratios and correlate this to pathological response.

^31^P‐MRSI was performed in 12 breast cancer patients treated with NAC. ^31^P spectra were fitted and aligned to the frequency of phosphoethanolamine (PE). Metabolic signal ratios for phosphomonoesters/phosphodiesters (PME/PDE), phosphocholine/glycerophosphatidylcholine (PC/GPtC), phosphoethanolamine/glycerophosphoethanolamine (PE/GPE) and phosphomonoesters/in‐organic phosphate (PME/Pi) were determined from spectral fitting of the individual spectra and the summed spectra before and after the first cycle of NAC. Metabolic ratios were subsequently related to pathological response. Additionally, the correlation between the measured metabolic ratios and Ki‐67 levels was determined using linear regression.

Four patients had a pathological complete response after treatment, five patients a partial pathological response, and three patients did not respond to NAC. In the summed spectrum after the first cycle of NAC, PME/Pi and PME/PDE decreased by 18 and 13%, respectively. A subtle difference among the different response groups was observed in PME/PDE, where the nonresponders showed an increase and the partial and complete responders a decrease (*P* = 0.32). No significant changes in metabolic ratios were found. However, a significant association between PE/Pi and the Ki‐67 index was found (*P* = 0.03).

We demonstrated that it is possible to detect subtle changes in ^31^P metabolites with a 7 T MR system after the first cycle of NAC treatment in breast cancer patients. Nonresponders showed different changes in metabolic ratios compared with partial and complete responders, in particular for PME/PDE; however, more patients need to be included to investigate its clinical value.

**Abbreviations used**
ATP: adenosinetriphosphate
ER: estrogen receptor
FID: free induction decay
GPC: glycerophosphocholine
GPE: glycerophosphoethanolamine
GPtC: glycerophosphatidylcholine
GPtE: glycerophosphatidylethanolamine
HER2: human epidermal growth factor receptor 2
NAC: neoadjuvant chemotherapy
PC: phosphocholine
PDE: phosphodiesters
PE: phosphoethanolamine
Pi: in‐organic phosphate
PME: phosphomonoesters
PR: progesterone receptor
SNR: signal‐to‐noise ratio

## 1 INTRODUCTION

Breast cancer is the most common cancer in women worldwide.^1^ Neoadjuvant chemotherapy (NAC) is increasingly used for presurgical treatment with the main purpose of downsizing locally advanced tumors to make them better candidates for resection.^2^ The recent St. Gallen consensus^3^ suggested a preference for NAC treatment in human epidermal growth factor receptor 2 positive (HER2+) subtypes and triple negative breast cancer, stage II and III. This preference was extended to women who were eligible for breast‐conserving surgery at diagnosis. It has been shown that the desired tumor size reduction is not accomplished with NAC in up to 31% of patients who will have stable or even progressive disease.^4^ In order to identify patients who will have stable or progressive disease, it is important to identify biomarkers that can assess the response to NAC at an early stage in the individual patient. This could enable the adjustment of systemic therapy and avoid toxicity and the related costs of ineffective treatment.

Currently, the effect of NAC during treatment is mainly quantified based on changes in tumor size upon clinical examination, using ultrasonography or MRI. Since underlying metabolic changes in response to treatment usually precede the relatively slow changes in tumor size,^5^, ^6^, ^7^, ^8^ monitoring of tumor metabolism may offer an earlier and better window to assess therapy response. Phosphorous magnetic resonance spectroscopic imaging (^31^P‐MRSI) of the breast makes it possible to assess changes in tumor metabolism. Metabolites of interest such as the phosphomonoesters (PME) (phosphocholine [PC] and phosphoethanolamine [PE]), and phosphodiesters (PDE) (glycerophosphocholine [GPC] and glycerophosphoethanolamine [GPE]), are known to be involved in anabolism and catabolism of the cell membrane.^9^, ^10^ Additionally, signals of mobile phospholipids glycerophosphatidylcholine (GPtC) and glycerophosphatidylethanolamine (GPtE), and metabolites involved in energy metabolism such as in‐organic phosphate (Pi) and adenosinetriphosphate (ATP), can be measured with ^31^P‐MRS.^11^, ^12^, ^13^

In the presence of cancer, elevated PME/PDE levels have been measured, where in most human tumors PE prevails over PC.^14^ In cases of an effective response to systemic therapy, a fall in PME to PDE levels is observed.^15^ In addition, it was shown that PME/PDE levels for triple negative tumors were low compared with estrogen/progesterone receptor positive (ER+/PR+) tumors.^16^, ^17^ Other studies demonstrated that ER‐ breast cancer is associated with low PC and high GPC concentrations,^18^ and that an overall reduction in PC and GPC concentration after the complete course of NAC treatment has been observed^19^ and is associated with long‐term survival.^20^

These previous studies were performed on tissue samples ex vivo with either proton MRS (^1^H‐MRS) or high resolution magic angle spinning MRS (HR MAS MRS) or in vivo at lower field strengths (up to 3 T). At 3 T, however, neighboring peaks with similar frequency (eg the phosphodiesters) are indistinguishable, and therefore moving up to 7 T is necessary. Studies have shown that ^31^P‐MRS at 7 T enables in vivo detection and quantification of phospholipid metabolites^21^, ^22^ with acceptable acquisition times and sufficient spatial resolution, particularly when using multi‐echo acquisitions.^23^

In previous work examining the feasibility of measuring in vivo changes in phospholipid metabolites during NAC using ^31^P‐MRSI,^24^ we reported decreased PME/PDE and PME/Pi halfway through and after the end of NAC. Therefore, the aim of this current study, in a different cohort of patients, was to assess the possibility of detecting these changes even earlier, directly after the first cycle of NAC using ^31^P‐MRS at 7 T, and to test the feasibility to differentiate pathological response levels to NAC.

## 2 MATERIALS AND METHODS

### 2.1 Patients

This MRI study was performed in accordance with the guidelines of the University Medical Center Utrecht ethics committee (trialregister.nl: NTR4980). Twelve breast cancer patients (mean age: 49 years; range: 36–64 years) gave informed consent to participate in this study. The patients were selected for treatment with NAC, and were examined with ^31^P‐MRSI before and after the first cycle of NAC (at ~3 week intervals). Table 1 summarizes the demographics and tumor characteristics of these patients.

**Table 1:** Demographics, tumor characteristics and pathological response of breast cancer patients undergoing neoadjuvant chemotherapy

**Patient**	**Age (y)**	**Treatment regime**	**ER**	**PR**	**HER2neu**	**TNM**	**PR** *	**Ki‐67 (%)**
1	56	6 x Taxotere – AC	−	−	−	T2N0M0	5	40
2	60	6 x Taxotere – AC	+	−	−	T2N0M0	4	10
3	64	6 x Taxotere – AC	+	+	−	T2N1M0	3	10
4	36	6 x Taxotere – AC	−	−	−	T2N0M0	2	40
5	62	4 x AC – 4 x taxotere with concomitant trastuzumab	+	−	+	T2N1M0	5	65
6	60	3 x FEC – 3 x docetaxel	+	+	−	T2N1M0	2	65
7	40	3 x FEC – 3 x docetaxel	+	−	−	T3N2M0	4	5
8	43	4 x AC – 4 x taxotere	−	−	−	T2N0M0	4	20
9	40	4 x AC – 12 x paclitaxel	−	−	−	T2N3M0	2	50
10	37	4 x AC – 4 x taxotere	+	+	−	T2N1M0	3	30
11	49	4 x AC – 4 x taxotere with concomitant trastuzumab	−	−	+	T2N0M0	5	35
12	44	4 x AC – 4 x taxotere with concomitant trastuzumab	−	−	+	T2N0M0	5	5

AC, adriamycin and cyclophosphamide; ER, estrogen receptor; FEC, 5‐fluorouracil, epirubicin and cyclophosphamide; HER2, human epidermal growth factor receptor 2; PR, progesterone receptor; TNM stage, classification of malignant tumors (tumor, nodes, metastasis).

* pathological response according to the Miller‐Payne system.

### 2.2 Pathology

All patients underwent surgery after completing NAC treatment and the pathological responses to NAC were defined according to the Miller‐Payne system^25^; complete response is classified as grade 5 and nonresponse as grade 1. Pathological nonresponse indicates no change or some alteration to individual malignant cells, but no reduction in overall cellularity compared with pretherapy core biopsy. Grades 1 and 2 were grouped together as nonresponders, grades 3 and 4 as partial responders, and grade 5 as complete responders. To investigate cell proliferation, immunohistochemistry for Ki‐67 was carried out in the pretherapy core biopsy, with a cut‐off point of 20%.^26^, ^27^, ^28^, ^29^

### 2.3 Acquisition

All patients were scanned in prone position with a unilateral two channel ^1^H/^31^P dual‐tuned breast coil (MR Coils, Zaltbommel, The Netherlands) on a 7 T MR system (Philips, Best, The Netherlands) without contrast agent. The scan session consisted of a fat suppressed T_1_‐weighted 3D MRI (TE = 2 ms, TR = 4 ms, flip angle = 10°, FOV = 160 x 160 x 160 mm^3^, isotropic resolution of 1.0 mm^3^). Fat suppression was achieved by using a short 1–2‐1 spectral‐spatial RF pulse. Third‐order B_0_ shimming was performed with least square error optimization using a 3D B_0_ with manual segmentation of the breast.^30^, ^31^P‐MRSI was obtained using the AMESING sequence,^23^ in which one FID and five full echoes were acquired within one TR, resulting in an FID at 0.45 ms and echoes at 45, 90, 135, 180 and 225 ms, respectively. Additional parameters were TR = 6 s, FOV = 160 x 160 x 160 mm^3^, 8 x 8 x 8 voxels, 2 x 2 x 2 cm^3^ nominal resolution, BW = 8200 Hz, and sampling matrix size = 256, resulting in a total scan time of 25:36 min for the ^31^P‐MRSI, excluding the standard imaging and shimming.

### 2.4 Data analysis

All MRSI data were analyzed using IDL 6.3 (Research Systems, Boulder, CO, USA), jMRUI 4.0^31^ and Matlab 2017a (MathWorks, Natick, MA, USA). Selection of the voxel containing the breast tumor was performed using in‐house Matlab scripts. All spectra were zero‐filled to 8192 data points and apodized (15 Hz Lorentzian) in the time domain and spatially Hamming‐filtered. To maximize the signal‐to‐noise ratio (SNR), the FID and five echoes were corrected for the difference in T_2_ relaxation of every metabolite for each individual patient. In a previous study, the T_2_ relaxation times of the different metabolites (nine in total) were determined based on the average spectra of that patient group.^32^ A spline interpolation between the T_2_ relaxation time and the chemical shift of the metabolites was used to calculate the weighted sum of the FID and five echoes.

After the T_2_‐weighting of the spectra, all spectra were frequency‐aligned to PE at 6.83 ppm. The individual metabolites were fitted in jMRUI using the AMARES algorithm.^31^ During the fitting, the overall phases were fixed to zero and the linewidth of PE had soft constraints with a minimum of 10 Hz and maximum of 40 Hz. The linewidths of PC, Pi and the diesters were set as a fixed ratio of the linewidth of PE. The chemical shift of PE was kept in the range of 6.79–6.89 ppm, the chemical shift of GPE in the range of 3.3–3.6 ppm, and the chemical shift of GPtC in the range of 2.25–2.35 ppm. Metabolic signal ratios for PME/PDE, PC/GPtC, PE/GPE and PME/Pi were calculated from the spectral fitting of the patients individually. To increase SNR even further and to observe general changes in the complete patient group, the spectra before and after the first cycle of NAC from all patients were summed separately. To investigate the differences among nonresponders, partial responders and complete responders, the spectra of the patients in the three groups were summed separately as well. The change in ratios (before NAC compared with after the first cycle of NAC) were determined and compared with the pathological response. Also, the mean PME/PDE ratio in triple negative tumors was compared with the mean PME/PDE ratio of the rest of the patients.

### 2.5 Statistical analysis

Statistical analysis was performed using an unpaired Mann–Whitney test (GraphPad Prism, GraphPad Software, San Diego, CA, USA), with a two‐tailed distribution to determine whether the metabolic ratios were significantly changed (α = 0.05) after the first cycle of NAC. For the correlation between the measured metabolic ratios before NAC treatment (baseline) and Ki‐67 levels, linear regression was performed and was considered statistically significant if *P* < 0.05.

A Kruskal‐Wallis test with a post‐hoc Dunn's multiple comparison test was used to assess statistical difference in metabolite ratios among the patient groups with different pathological responses (nonresponders, partial responders and complete responders).

## 3 RESULTS

Three patients had a HER2+ tumor and the other patients had HER2‐ tumors. Six tumors were ER+, three of which were PR+, and four triple negative tumors were included in this study. All patients completed all cycles of NAC; four patients had a pathological complete response after treatment, five patients had a partial pathological response, and three patients showed no response (Table 1). All three HER2+ subtypes had a complete response to NAC treatment.

**Figure 1:**
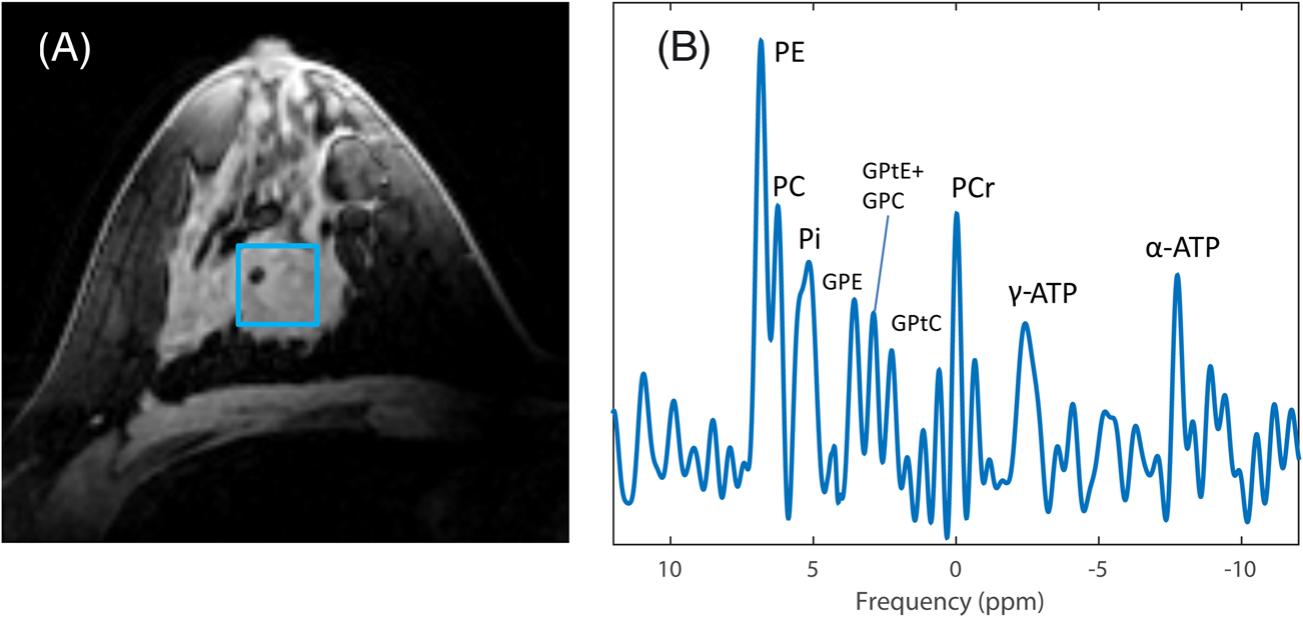
Example data of a patient with an ER+, PR+, HER2‐ tumor. A, T_1_ weighted 3D FFE image with a representation of the selected voxel (blue square) for ^31^P analysis. B, the corresponding T_2_ weighted spectrum shows all nine fitted metabolites

**Figure 2:**
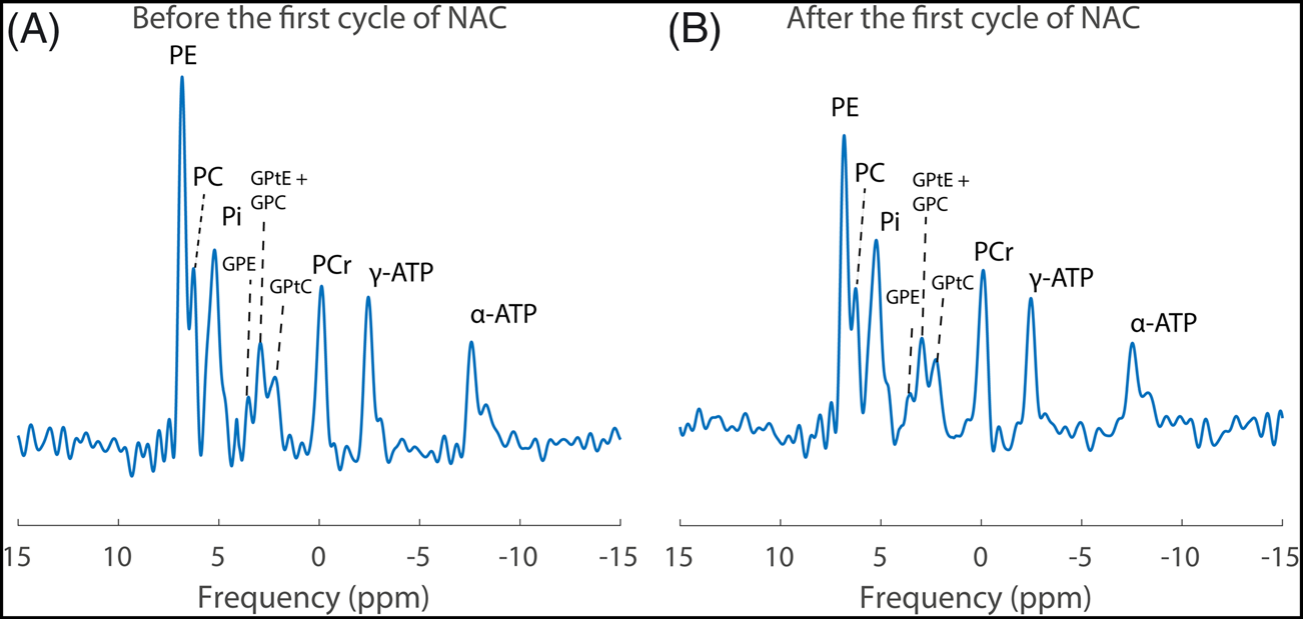
Summed spectrum of all patients A, before and B, after the first cycle of NAC with all fitted ^31^P metabolites clearly visible

After selecting the voxel location in the middle of the tumor (Figure 1), the spectrum clearly showed all nine metabolites, including all three peaks of PDE. In the summed spectra for all patients (Figure 2), a broad Pi peak compared with the other metabolites was observed. After the first cycle of NAC, PME/Pi and PME/PDE decreased by 18 and 13%, respectively.

**Figure 3:**
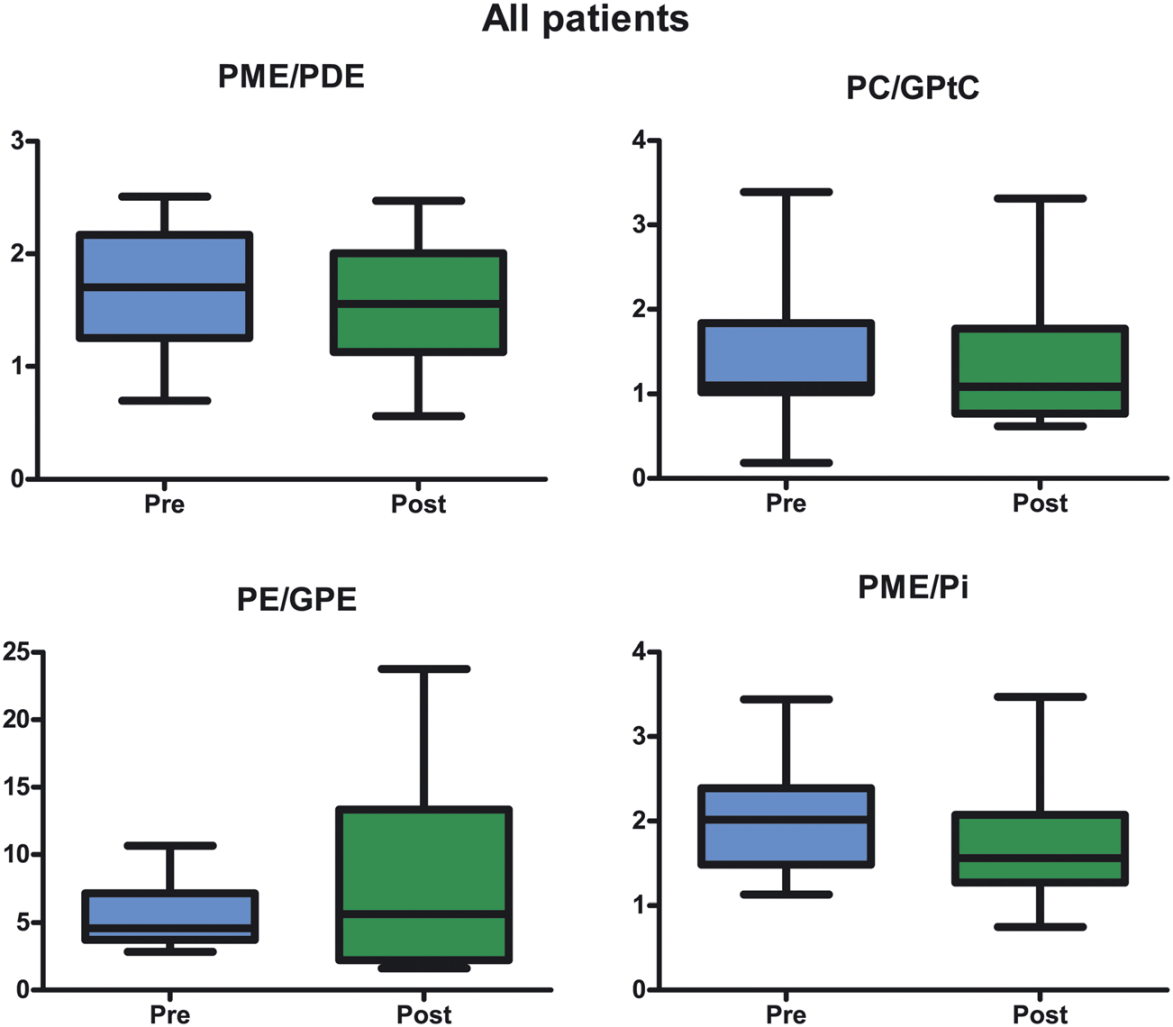
Whisker boxplot with 5–95 percentile of the mean metabolic ratios of different metabolites before (blue) and after (green) the first cycle of neoadjuvant chemotherapy in all patients (including all different kinds of response). All ratios show a decrease except for PE/GPE that shows an increase. However, note the high standard deviation of PE/GPE, indicating an outlier where a very large increase in this ratio was observed

Metabolic ratios involved in membrane and energy metabolism were calculated for all patients. No significant differences were found between the metabolic ratios measured before and after the first cycle of NAC. For all patients, PME/Pi before the start of NAC was >1 and decreased after the first cycle of NAC (Figure 3).

**Figure 4:**
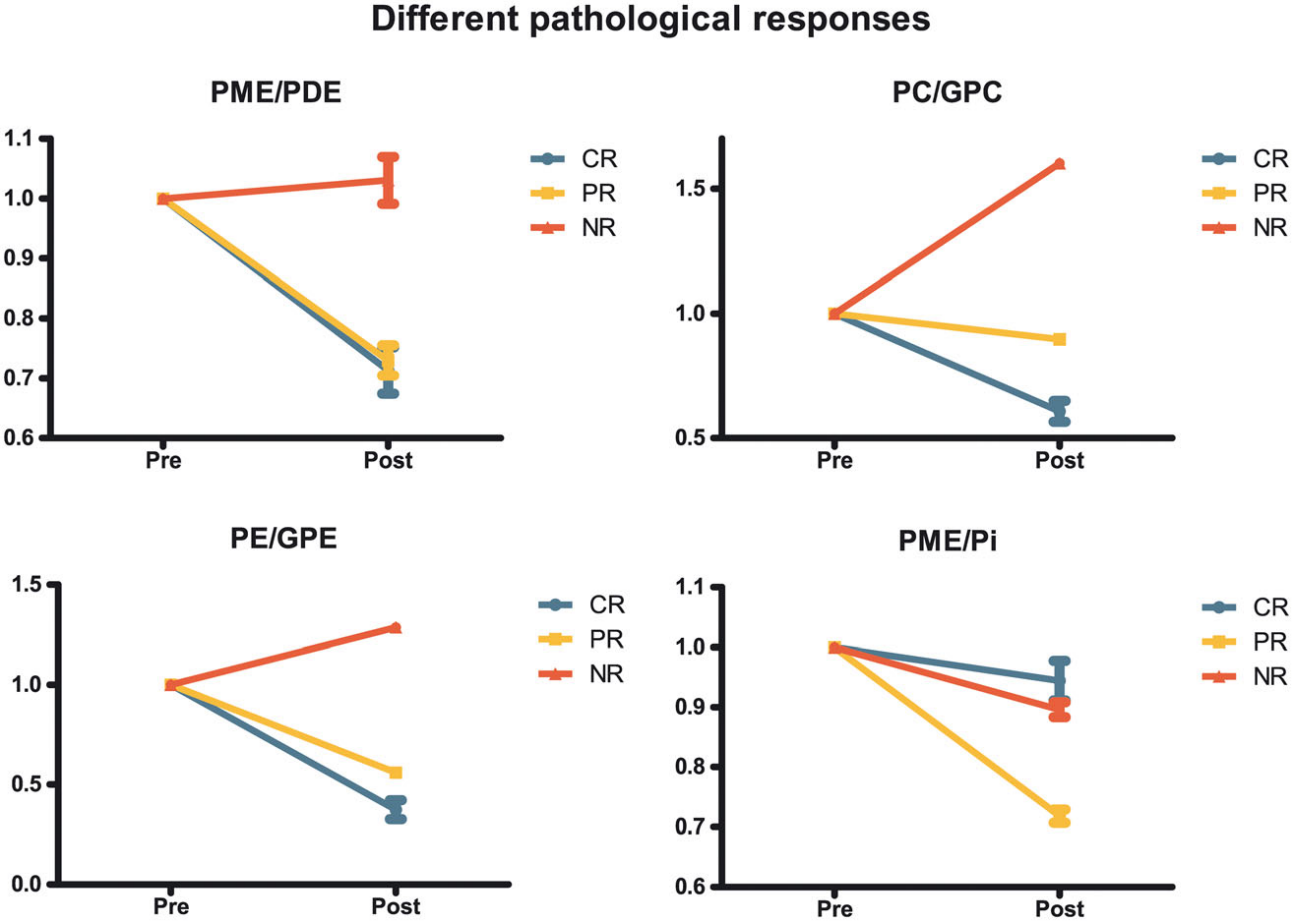
Changes in metabolic ratios with standard deviation before (pre) and after (post) the first cycle of neoadjuvant chemotherapy for the different pathological responses (CR, complete responders; PR, partial responders; NR, nonresponders) where the ratios before NAC are normalized to one. This data was obtained by spectral fitting of the summed spectra of the different pathological responses. Note that the direction of the change in PME/PDE for the nonresponders is in an opposite direction compared with the complete and partial responders. The shown standard error is the fitting error and not the deviation among different patients

When comparing metabolic ratios of the three pathological responses groups (Figure 4), the largest difference was observed in PME/PDE. The nonresponders showed an increase, and the partial and complete responders a decrease (*P* = 0.32). The change in PC/GPtC suggests that this ratio has a more linear distribution among the different pathological responses, showing the largest increase for the nonresponders, no change to a small increase for the partial responders, and a decrease of the ratio for the complete responders.

**Figure 5:**
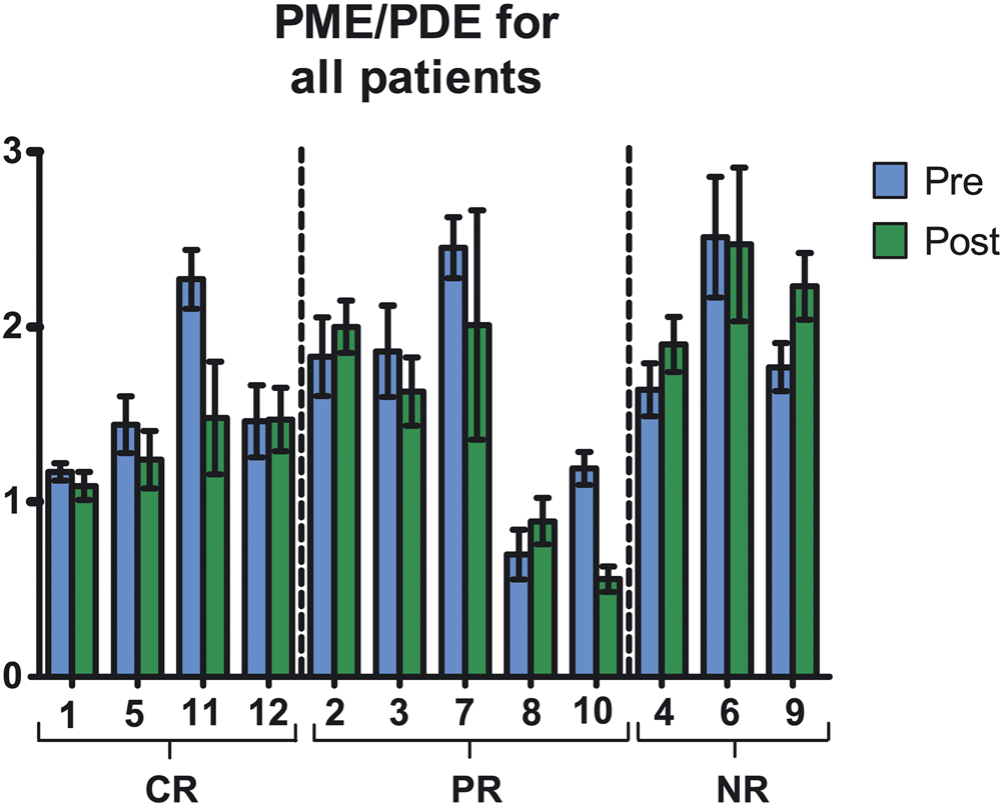
PME/PDE measured after spectral fitting of the individual ^31^P spectra before (blue) and after (red) the first cycle of NAC arranged for the different pathological responses. The numbers below the graphs correspond to the patient numbers in Table 1 and the standard deviation is the standard error of the fit. In some cases (patients 1, 2, 3 and 11), GPE could not be fitted

For PME/PDE we had a closer look (Figure 5) to see if the results still held for the individual fits instead of the summed spectra of the different response groups. Two of the three nonresponders showed an increase in the ratio while the other nonresponders showed only a small decrease. However, two of the five partial responders also showed an increase of PME/PDE. Three out of four complete responders showed a decreased PME/PDE; however, no significant difference was found (*P* = 0.89).

**Figure 6:**
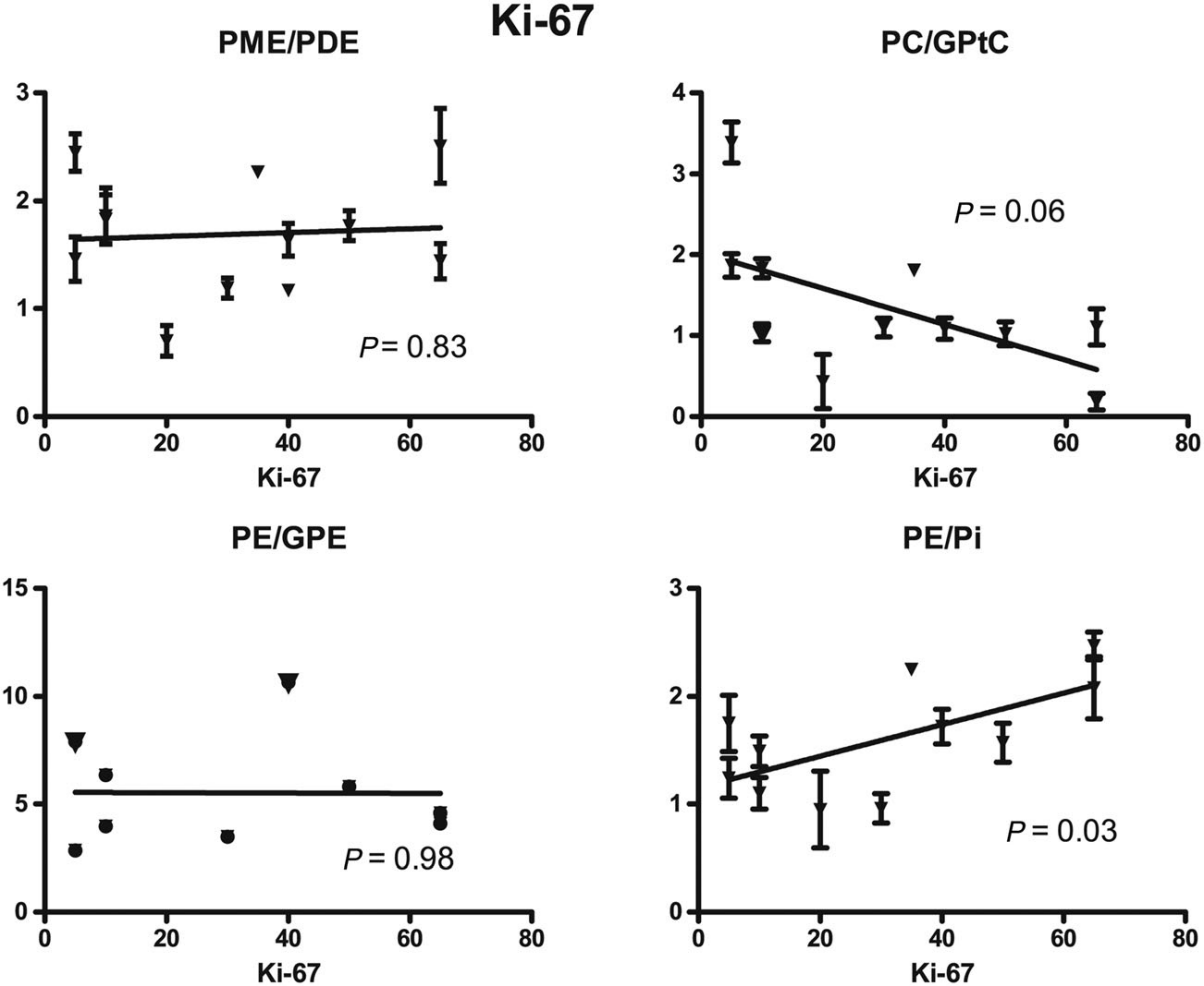
The different metabolic ratios of all patients before NAC treatment against Ki‐67 index with the corresponding *P*‐values

All metabolic ratios were correlated with the Ki‐67 index (Figure 6). A significant statistical association between PE/Pi and the Ki‐67 index was observed (*P* = 0.03); all other ratios were not significantly correlated with the Ki‐67 index. The Kruskal‐Wallis test with the post‐hoc Dunn's multiple comparison test showed no significant differences among the three different pathological responses for the metabolic ratios after the first cycle of NAC.

## 4 DISCUSSION

The potential of using ^31^P‐MRSI for treatment response assessment in tumors has been shown in previous studies.^9^, ^24^, ^33^, ^34^, ^35^, ^36^ In this preliminary study, we demonstrated that changes in phospholipid metabolites can be detected after the first cycle of NAC and that these changes might be related to the different pathological responses. For PME/PDE, the group of nonresponders showed an increased ratio after the first cycle of NAC treatment while the partial and complete responders showed a decrease of this ratio. However, when we looked at the individual fits of PME/PDE, two of the five partial responders showed an increase in this ratio. The main reason for this discrepancy is probably the lower SNR for the individual spectra compared with the summed spectra of the different groups. In some of the metabolic ratios (PME/PDE and PME/Pi) a large variation among patients was observed, which is probably due to physiological differences in the heterogeneous patient group of our study including different kinds of tumors and different kinds of NAC treatment. It has been shown in several studies that estrogen and progesterone can be involved in activation of choline‐kinase.^16^, ^37^ However, in this study no correlations were found among the metabolic ratios and ER status and PR status, which is probably due to the relatively low number of patients included. A larger patient population is required for a more comprehensive analysis of change in the different metabolic ratios in relation to the pathological response, which, due to Food and Drug Administration clearance on 7 T MRI, is expected to become a reality in the near future. Additionally, no contrast agent was used during acquisition, theoretically enabling the possibility to restage the tumor several times during treatment, which is an advantage for the patient given the controversy over the use of gadolinium.^38^

A linear relationship was found in this study between PE/Pi before NAC and the Ki‐67 index. This was expected, as PE is an important metabolite involved in the membrane metabolism and the Ki‐67 index is a marker for cell proliferation, which are closely related to each other. In the summed spectra of all patients, however, no significant differences in the metabolite levels were found between the two measurements. A possible explanation could be that in the summed spectra all pathological responses are included. The observed decreases in PME/PDE and PME/Pi after the first cycle of NAC correspond with findings in a previous study that measured these metabolite ratios after three cycles of NAC.^24^

In line with another study,^15^ high PME/PDE in the tumor tissue were observed in our study, with a mean ratio of 1.69 compared with healthy volunteers (mean ratio of 0.70^32^). Preclinical work and animal models^16^, ^17^ have shown that PME/PDE for triple negative tumors are low compared with ER+/PR+ tumors. In the present study we showed that these findings still hold for measurement in breast cancer patients, with a mean PME/PDE for triple negative tumors of 1.32 ± 0.42 compared with a mean PME/PDE of 1.88 ± 0.46 for the other patients.

The PME/Pi is a tissue viability marker that may be used as an early marker for tumor cell apoptosis in radiation therapy.^39^ Also, in hepatic lymphomas, increased levels of PME/Pi were observed, which decreased after admission of chemotherapy. It was concluded that the drugs reached the target cells and affected tumor cell metabolism or killed the tumor cells.^40^ In all patients in this study, PME/Pi was >1 and decreased after the first cycle of NAC, supporting the findings in a previous study in breast cancer patients at 7 T,^24^ and in line with studies in other settings. However, caution is needed when drawing conclusions from this ratio in breast cancer patients. If the tumor is closely situated near the pectoral muscle, the PME/Pi can be contaminated with the Pi signal originating from the pectoral muscle.

The broadening of the Pi peak, as compared with the other metabolites, is possibly related to pH differences among tumors, but also to pH changes during chemotherapy. As the chemical shift of Pi is sensitive to pH changes, a broadening is expected when summing the patients' spectra before and after NAC treatment. The spectra of the different pathological responses also showed broadening of the Pi peak, suggesting that the Pi shift is not dependent on the pathological response to the therapy.

We were able to perform analysis for every individual patient; however, in some patients one of the metabolites could not be fitted (GPE in most cases). This was mainly due to low SNR, which makes the fit of overlapping peak unreliable, particularly for the phosphodiesters. This resulted in some outliers, for example, in PC/GPtC and PE/GPE. To gain more SNR we decided to sum the spectra for every different pathological response resulting in good spectra where all nine metabolites were clearly visible. The SNR of the ^31^P MR spectra is low even at 7 T, which indicates that translation of the technology to more widely available 3 T MRI systems may be out of reach. However, technologies like polarization transfer^41^ and local ^31^P receiver arrays^42^ with uniform transmit^43^ are expected to improve SNR, which could be translated to reduce scan times or improve accuracy in the detection of the ^31^P metabolites. A body coil will generate a more homogeneous transmit field, making adiabatic pulses unnecessary, enabling shorter TR and, via Ernst angle excitations, an improved SNR in the same scan time. The use of local receive array coils could even speed up the acquisition by using parallel imaging techniques.

## 5 CONCLUSION

We demonstrated that changes in ^31^P metabolites can be detected by 7 T MRI after the first cycle of NAC in breast cancer patients. Modest changes among patient groups with different pathological responses were detected after the first cycle of NAC based on the different metabolic ratios. PME/PDE showed the highest potential to discriminate nonresponders from partial and complete responders, yet warrant the inclusion of more patients to potentially reach significance.

## FUNDING INFORMATION

The Dutch Cancer Society and the NWO provided financial support for this study.
